# miR-200a-3p- and miR-181-5p-Mediated HOXB5 Upregulation Promotes HCC Progression by Transcriptional Activation of EGFR

**DOI:** 10.3389/fonc.2022.822760

**Published:** 2022-06-29

**Authors:** Weizhi Li, Yingchao Li, Peijie Li, Fuquan Ma, Mengying Liu, Shuzhen Kong, Hui Xue

**Affiliations:** ^1^Department of Gastroenterology, The First Affiliated Hospital of Xi’an Jiaotong University, Xi’an, China; ^2^Department of Dermatology, The Second Affiliated Hospital of Xi’an Jiaotong University, Xi’an, China

**Keywords:** HCC, HOXB5, miR-200a-3p, miR-181-5p, EGFR

## Abstract

**Background:**

Hepatocellular carcinoma (HCC) remains a worldwide burden. However, the mechanisms behind the malignant biological behavior of HCC remain unclear. The homeobox (HOX) family could act as either promoters or suppressors in different kinds of malignancies. Our study discovered the role of HOXB5 in regulating HCC progression.

**Methods:**

The HOXB5 expression was assessed by RT-PCR analysis in human HCC samples and cell lines. HOXB5 transcriptional regulation of the EGFR was verified by the luciferase reporter assay and chromatin immunoprecipitation experiment. The oncogenic role of HOXB5 in HCC progression was analyzed by CCK8, colony-forming, and transwell assays.

**Results:**

Upregulation of HOXB5 was found in human HCC, and was strongly correlated with HCC tumor size, tumor-nodule metastasis, TNM stage, and relatively unfavorable OS and DFS. Ectopic expression of HOXB5 promoted the capacity of cell growth and clonogenicity, while the inhibition of HOXB5 decreased the proliferation and clonogenicity potential *in vitro* by CCK8 and colony-forming assays. In addition, HOXB5 also promoted cell migration by transwell experiment. Mechanism studies elucidated that HOXB5 triggers HCC progression *via* direct transcriptional activation of EGFR. The upregulation of HOXB5 is regulated by miR-200a-3p and miR-181-5p. Transfection of miR-200a-3p and miR-181-5p mimics blocked the cell proliferation and migration regulated by HOXB5, while overexpression of the 3′-UTR mutant HOXB5 abolished the suppressive effect of miR-200a-3p and miR-181-5p, but not the wild-type HOXB5.

**Conclusion:**

HOXB5 is a promising prognostic factor in human HCC. Targeting miR-200a-3p and the miR-181-5p/HOXB5/EGFR signaling pathway may provide new options for the treatment strategies of HCC.

## Introduction

Despite the fact that significant progress has been made in medicine and treatment, hepatocellular carcinoma (HCC) remains a worldwide burden due to its high morbidity and mortality, and a relatively poor 5-year survival rate (1). A wide variety of basic and clinical investigations of HCC have been made every year, and mountains of molecules and related signaling pathways had been reported every year (2, 3). Unfortunately, the mechanisms behind the malignant biological behavior of HCC remain elusive; thus, it is important to seek for such hidden mechanisms.

Elevation of the epidermal growth factor receptor (EGFR) is frequently found in metastatic and recurrence tumors, especially in HCC. In recent years, anti-EGFR therapy has achieved tremendous success in solid tumors of the digestive system, such as colorectal cancer (4). Logically, targeting EGFR could be an intriguing proposal for HCC treatment, given that EGFR is extensively expressed in both hepatocytes and mesenchymal cells of the liver. However, anti-EGFR therapy failed to meet expectations in HCC treatment. So far, the underlying mechanisms remain elusive. Thus, targeting the upstream regulators of EGFR brings the attention to HCC therapy. The homeobox (HOX) superfamily, which is famous for its role in tumor progression, is often dysregulated in tumor development *via* interfering apoptosis, proliferation, differentiation, motility, and angiogenesis (5). It is reported that the HOX family could act as either promoters or suppressors in different kinds of malignancies. HOXB5 is a key family member of HOX that has been proved to exhibit an oncogenic role in the initiation and progression of tumors (6–8). HOXB5 regulated cancer cell proliferation *via* different signaling pathways, mainly including Wnt/β-catenin and Akt pathways (9, 10). HOXB5 is involved in tumor metastasis mainly through inducing matrix metalloproteinase (MMP) production and facilitating epithelial-to-mesenchymal transition (EMT) (9, 11). HOXB5 is also a master target of multiple cancer-related microRNAs (miRNAs) (12, 13), further proving the central role of HOXB5 in cancer development. A previous study outlined the role of HOXB5 in HCC proliferation, but there is a lack of in-depth mechanism studies. Yet, whether HOXB5 is involved in HCC metastasis remains unknown.

In our study, to clarify the role of HOXB5 in regulating HCC progression, we tested the expression status of HOXB5 in HCC tissues from surgery patients and confirmed the upregulation of HOXB5 in HCC tissues compared with adjacent controls. A higher level of HOXB5 was associated with HCC size, HCC-nodule metastasis, TNM stage, and relatively unsatisfied overall survival (OS) and disease-free survival (DFS). Then, we established the overexpression and downregulation HCC cell lines of HOXB5 to seek the functions and mechanisms of HOXB5 in HCC progression. Our study demonstrated that ectopic expression of HOXB5 in HepG2 cells promoted the capacity of cell growth and clonogenicity, while the inhibition of HOXB5 in Hep3B cells decreased the proliferation and clonogenicity potential *in vitro* by CCK8 and colony-forming assays. In addition, HOXB5 also promoted cell migration *in vitro* by transwell experiment. Mechanism studies elucidated that the upregulation of HOXB5 is regulated by miR-200a-3p and miR-181-5p. Transfection of their mimics reduced the cell proliferation and migration regulated by HOXB5, while overexpression of the 3′-UTR mutant HOXB5 abolished the suppressive effect. In addition, we found that HOXB5 triggers HCC progression *via* direct transcriptional activation of EGFR.

## Materials and Methods

### Ethics Statement

All the human samples were obtained during surgical treatment at the First Affiliated Hospital of the Xi’an Jiaotong University, which was under the supervision of the Clinical Research Ethics Committee. The patients enrolled in our research were free of other types of therapy before the surgery, and all patients who donated tissue samples provided written informed consent.

### Patients and Tissue Samples

One hundred paired primary and adjacent non-tumor samples of HCC were obtained during surgical treatment at the First Affiliated Hospital of the Xi’an Jiaotong University (Xi’an, China), which was under the supervision of the Clinical Research Ethics Committee. The patients enrolled in our research were free of other types of therapy before the surgery, and all patients who donated tissue samples provided written informed consent.

### Cell Culture and Treatments

The human HCC cell lines HepG2, Huh-7, and Hep3B were commercially available from the Cell Bank of the Chinese Academy of Sciences (Shanghai, China). The obtained HCC cells were cultured in DMEM (Gibco, USA) combined with 10% fetal bovine serum (FBS) in a standard atmosphere with 5% CO_2_. miR-mimics and inhibitors were obtained from GenePharma Biotechnology (Shanghai, China). For the transfection procedure, overexpression and downregulation cells of HOXB5 were generated with LV-HOXB5 and *sh*-HOXB5 by lentivirus vectors. The transfection of 100-μm mimics or inhibitors and their corresponding negative controls was carried out using Lipofectamine 2000 reagent (Invitrogen, USA).

### Protein Preparation and Western Blot

The whole proteins of tissues and cells were treated with RIPA buffer, protease, and phosphatase inhibitors (Merck Millipore, USA). The concentrations of each protein were measured by the BCA assay kit (Beyotime, Shanghai, China), and proteins were separated by SDS/PAGE at a loading concentration of 30 μg. The proteins were then transferred to a polyvinylidene fluoride (PVDF) membrane (Merck Millipore, USA) and incubated with primary antibodies at 4°C overnight, followed by incubation with secondary antibodies for 1 h at room temperature (RT). The primary antibodies were as follows: anti-β-actin (Sigma, USA) anti-HOXB5 (Bioss, China).

### RNA Isolation and Real-Time PCR Analysis

The total RNA was extracted from 30 pairs of fresh frozen HCC and adjacent normal tissues by TRIzol reagent (Invitrogen). cDNA was obtained by reverse transcription. The expression levels of the determined mRNAs and miRNAs were measured using quantitative real-time PCR (Bio-Rad, Hercules, CA, USA). The mRNA values were normalized to β-actin expression before comparison, while miRNA values were normalized to U6 expression before comparison.

### *In Vitro* Functional Studies

Cell proliferation studies were evaluated by the CCK8 assay and colony-forming assay. The CCK8 assay was performed by Cell Counting Kit-8 kit (Dojindo, Kumanoto, Japan), and cells were plated into 96-well plates at a concentration of 3,000 cells/well after different treatments. Then, the cells were incubated with CCK8 solution for 4 h, and cell viability was evaluated by measuring the absorbance at 490 nm every day for 5 days. The colony-forming assay was performed by seeding cells (1,000–1,500/well) into six-well plates for 14 days after different treatments. Then, the cells were fixed with absolute ethyl alcohol and dyed with 5% crystal violet. The cell migration assay was evaluated by transwell. The migration assay was performed by directly seeding cells (5 × 10^4^) into the upper Boyden chambers (Corning, USA). The upper chambers were filled with DMEM medium (containing 1% FBS), while lower chambers were filled with DMEM medium (containing 20% FBS). After culturing for 24 h, the cells adhering to the lower side of the membrane were fixed and stained with 0.5% crystal violet, and the number of cells in each well was calculated under 5 fields with an optical microscope.

### Dual-Luciferase Reporter Assay

The effects of miR-200a-3p and miR-181-5p on HOXB5 activity were measured by dual-luciferase reporter assays. The HepG2 cells were transfected with either the wild-type (WT) or mutant (MUT) 3′-UTR of HOXB5, which was inserted into the pGL3 vector in 96-well plates, after co-transfecting with miR-200a-3p or miR-181-5p mimics into HCC cells using Lipofectamine 2000 for 24 h; the dual-luciferase assays were performed using the dual-luciferase reporter assay system (Promega, Madison, WI, USA).

### Chromatin Immunoprecipitation Assay

Cells were cultured to 80%–90% confluency before being harvested. ChIP assay was performed according to the manufacturer’s instructions. DNA samples were analyzed by real-time quantitative PCR.

### Statistical Analysis

The statistical results were evaluated by using SPSS 22.0 software (Chicago, IL, USA). The quantitative data are presented as the mean ± SD. The Student’s unpaired *t*-test was used to compare the differences between the two groups, while the one-way ANOVA was used to make comparisons between the control group and several experimental groups. The paired *t*-test was used to assess the expression of miR-200a-3p or miR-181-5p and HOXB5 between HCC tissues and the corresponding adjacent normal tissues, and the relationship between miR-200a-3p or miR-181-5p and HOXB5 was calculated using the chi-square test. All *p*-values were two-sided, and *p* < 0.05 was considered statistically significant.

## Results

### Elevated Expression of HOXB5 Is Frequently Found in HCC Patients and Positively Correlated With Unsatisfied Prognosis

**Figure 1:**
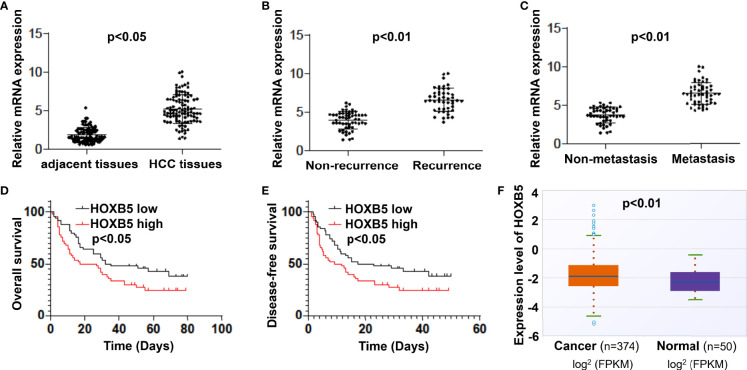
Elevated expression of HOXB5 is frequently found in HCC patients and positively correlates with poor prognosis. **(A)** mRNA expression level of HOXB5 in 100 pairs of HCC tissues and adjacent non-tumor tissues was evaluated by qRT-PCR. Data are mean ± SD, *n* = 100. **(B)** mRNA levels of HOXB5 were significantly higher in recurrence patients than in patients free of recurrence. **(C)** The HOXB5 expression level was much higher in metastasis patients than in patients free of metastasis. **(D, E)** Kaplan–Meier curve of HOXB5 depicting the OS and DFS of HCC patients. **(F)** mRNA expression of HOXB5 in human HCC was evaluated in a public database.

Abnormal expression of HOXB5 human cancers, including that in HCC, had been reported previously. To verify the expression status of HOXB5 in HCC, especially its important clinical implications, we identified the mRNA expression level of HOXB5 in 100 pairs of HCC tissues and adjacent non-tumor tissues. The expression of HOXB5 was higher in HCC tissues than in their adjacent non-tumor tissues (**Figure 1A**, **Table 1**). Moreover, the mRNA level of HOXB5 was significantly higher in recurrence HCC patients than in patients free of recurrence (**Figure 1B**). Moreover, the HOXB5 expression level was much higher in metastasis patients than in patients without metastasis (**Figure 1C**). Elevated HOXB5 expression was positively correlated with tumor size, tumor-nodule metastasis, and TNM stage (**Table 1**). Patients with higher HOXB5 expression predict a relatively unfavorable OS and DFS (**Figures 1D, E**). We also evaluated mRNA expression of HOXB5 in human HCC in a public database and found increased HOXB5 expression in HCC tissues compared with normal tissues (**Figure 1F**). In all, the results suggested that HOXB5 is upregulated in HCC and acted as a prognostic factor of HCC patients.

**Table 1:** Correlation between HOXB5 expression and clinicopathological characteristics of HCC.

Clinicopathological variables	Total	HOXB5		*p*
		Low	High
**Sex**				0.34
Male	23	9	14	
Female	77	22	55	
**Age (years)**				0.79
≤50	52	17	35	
>50	48	14	34	
**Serum AFP**				0.03
≤20 ng/ml	35	11	24	
>20 ng/ml	65	20	45	
**HBV infection**				0.04
Negative	37	15	22	
Positive	63	16	47	
**Child-pugh score**				<0.001
Class A	79	24	55	
Class B	21	7	14	
**Tumor size**				0.04
≤5 cm	74	20	54	
>5 cm	26	11	20	
**Tumor-nodule metastasis**				0.01
Absent	31	17	14 0	
Present	69	14	55	
**TNM stage**				<0.001
1/11	67	28	39	
II	33	3	30	

### HOXB5 Promotes Cell Proliferation and Accelerates Migration

**Figure 2:**
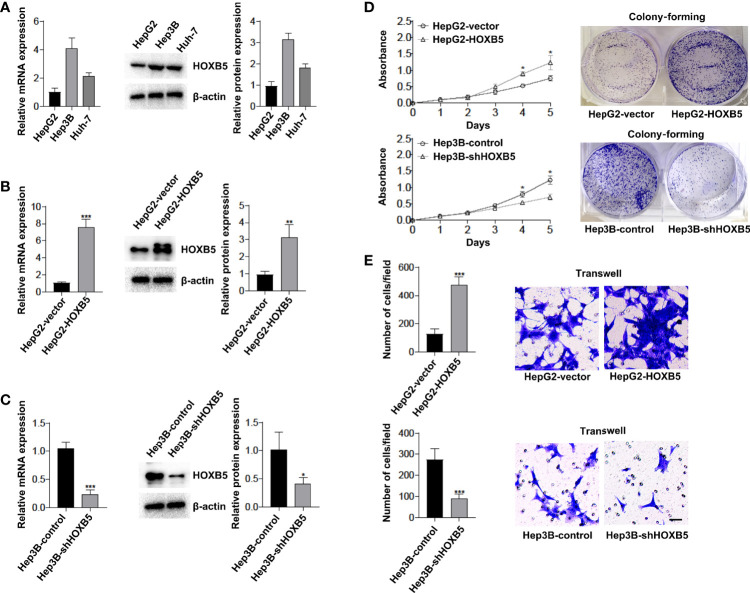
HOXB5 promotes cell proliferation and migration of HCC cells. **(A)** Expression of HOXB5 in HepG2, Huh-7 and Hep3B HCC cell lines as tested by qRT-PCR and Western bolt. Data are mean ± SD. **p < 0.01, ***p < 0.001; **(B, C)** Expression of HOXB5 in HepG2-HOXB5 and Hep3B-shHOXB5, and their corresponding control cell lines as tested by qRT-PCR and Western bolt. Data are mean ± SD. **p < 0.01, ***p < 0.001; **(D)** CCK8 and clonogenicity assays of proliferation capability of HCC cells. Data are mean ± SD. *p < 0.05, ***p < 0.001; **(E)** Invasion and migration assays of HepG2-HOXB5 and Hep3B-shHOXB5, and their corresponding control cell lines as tested by transwell experiment. Data are mean ± SD. *p <0.05. Scale bars, 100µm.

To determine the functional role of HOXB5, we generated the overexpression and downregulation cell lines, HepG2-HOXB5 and Hep3B-*sh*HOXB5, and their corresponding control cell lines according to the expression level of HOXB5 in HepG2, Huh-7, and Hep3B cell lines (**Figure 2A**). Ectopic expression or silencing of HOXB5 was confirmed by RT-PCR and Western blot analysis (**Figures 2B, C**). We found that overexpression of HOXB5 significantly increased cell viability and clonogenicity in HepG2 cells (**Figures 2D, E**). Conversely, silencing of HOXB5 restricted cell viability and clonogenicity in Hep3B cells (**Figure 2D**). The pro-migration effect of HOXB5 was validated by transwell assay. Ectopic expression of HOXB5 accelerated cell migration in HepG2 cells, while silencing of HOXB5 restricted the ability of Hep3B cells to migrate (**Figure 2E**). Thus, HOXB5 exerted its oncogenic function in HCC progression *via* promoting cell proliferation and accelerating migration.

### EGFR Is a Directly Transcriptional Target of HOXB5

**Figure 3:**
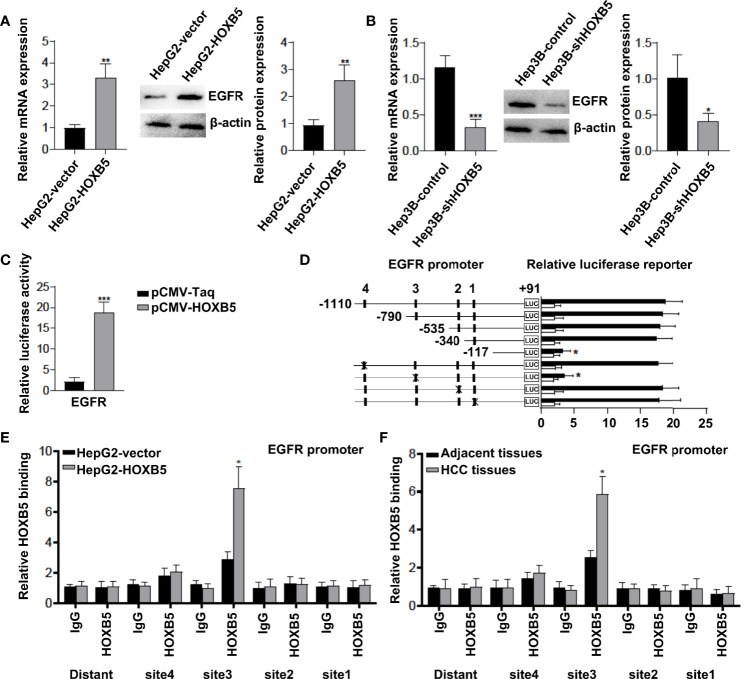
EGFR is a direct transcriptional target of HOXB5. **(A, B)** Expression of EGFR in HepG2-HOXB5 and Hep3B-*sh*HOXB5, and their corresponding control cell lines as tested by qRT-PCR and Western blot. Data are mean ± SD. *p < 0.05, **p < 0.01, ***p < 0.001. **(C)** The luciferase reporter assay demonstrated that ectopic expression of HOXB5 promoted the luciferase activity of the EGFR promoter. **(D)** EGFR promoter. Serially truncated and mutated EGFR promoter constructs were co-transfected with pCMV-HOXB5, and relative luciferase activities were determined. **(E, F)** ChIP assays demonstrated the direct binding of HOXB5 to the EGFR promoter in HepG2-HOXB5 cells and the enriched binding of endogenous HOXB5 to the EGFR promoter in primary HCC tissues.

Previous studies revealed that EGFR is one of the genes mainly regulated by HOXB5 in cancer progression (14). Considering that EGFR is predominantly expressed in hepatocytes, and significantly enriched when HCC occurs, we hypothesized that EGFR may be regulated by HOXB5 in HCC. To confirm our hypothesis, we first analyzed the expression of EGFR in HepG2-HOXB5 and Hep3B-*sh*HOXB5, and their corresponding control cell lines. As expected, upregulation of HOXB5 led to increased expression of EGFR mRNA and protein expression, while EGFR expression in Hep3B-*sh*HOXB5 cells was significantly decreased compared with control (**Figures 3A, B**). The luciferase reporter assay demonstrated that ectopic expression of HOXB5 increased the luciferase activity of the EGFR promoter (**Figure 3C**). We then examined and analyzed the direct binding of HOXB5 to the promoter region of EGFR in HepG2 cells stably overexpressing HOXB5 and control cells, and the reporter assay showed that depletion of the cis-element located between −790 and −625 significantly reduced the activity of the EGFR promoter mediated by HOXB5 overexpression, while mutation of the putative HOXB5 binding sites in this fragment decreased HOXB5-mediated activation of the EGFR promoter (**Figure 3D**). Furthermore, the ChIP assay further demonstrated that HOXB5 binding was indeed enriched in these regions in both HepG2-HOXB5 cell lines and human HCC tissues (**Figures 3E, F**). Collectively, these data indicated that EGFR is a direct transcriptional target of HOXB5 in HCC.

### HOXB5 Promotes HCC Progression by Upregulating EGFR Expression

**Figure 4:**
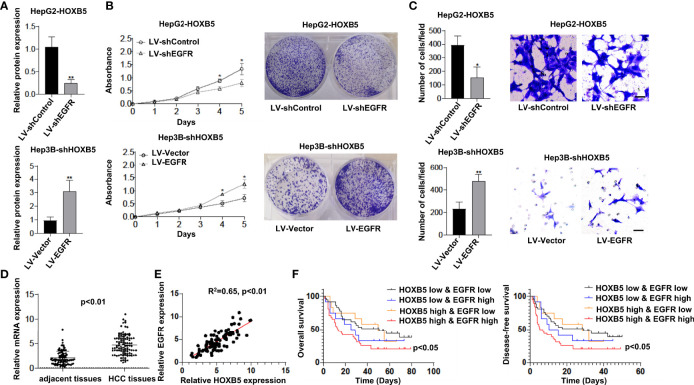
HOXB5 promotes HCC progression by upregulating EGFR expression, and positively correlated with EGFR expression in human HCC tissues. **(A)** Expression of EGFR in HepG2-HOXB5 and Hep3B-shHOXB5, and their corresponding control cell lines as tested by Western bolt. Data are mean ± SD. **p < 0.01; **(B)** CCK8 and clonogenicity assays of proliferation capability of HCC cells. Data are mean ± SD. *p < 0.05; **(C)** Migration assay of HepG2-HOXB5 and Hep3B-shHOXB5, and their corresponding control cell lines as tested by transwell experiment. Data are mean ± SD. *p < 0.05, **p < 0.01. Scale bars, 100µm; **(D)** mRNA expression level of EGFR in 100 pairs of HCC tissues and adjacent non-tumor tissues was evaluated by qRT-PCR. Data are mean ± SD, n=100; **(E)** EGFR expression is positively corelated with HOXB5 expression; **(F) **Kaplan-Meier analysis showed that HCC patients with positive co-expression of EGFR and HOXB5 showed lowest OS and DFS times.

To determine whether EGFR is involved in HOXB5-mediated HCC progression, we knocked down EGFR expression in HepG2-HOXB5 cells (**Figure 4A**). CCK8 and colony-forming assays showed that knockdown of EGFR expression restricted cell viability and clonogenicity in HepG2-HOXB5 cells (**Figure 4B**). Consistently, the transwell assay revealed that knockdown of EGFR expression dramatically reversely restricted the migration capacity mediated by HOXB5 overexpression (**Figure 4C**). These studies suggest that HOXB5 promoted HCC progression by upregulating EGFR expression.

### HOXB5 Expression Is Positively Correlated With EGFR Expression in Human HCC Tissues

To further validate the potential correlation between HOXB5 and EGFR, we first analyzed the mRNA expression of EGFR in tissues of human HCC. We found a significant upregulation of EGFR in HCC tissues than in their adjacent non-tumor tissues (**Figure 4D**). More importantly, EGFR expression is positively correlated with HOXB5 expression (**Figure 4E**). Furthermore, Kaplan–Meier analysis showed that HCC patients with positive co-expression of EGFR and HOXB5 showed the lowest OS and DFS times (**Figure 4F**). These findings suggested that HOXB5 is an important upstream regulator of EGFR in HCC patients.

### miR-200a-3p and miR-181-5p Upregulate HOXB5 Expression to Contribute to HCC Progression

**Figure 5:**
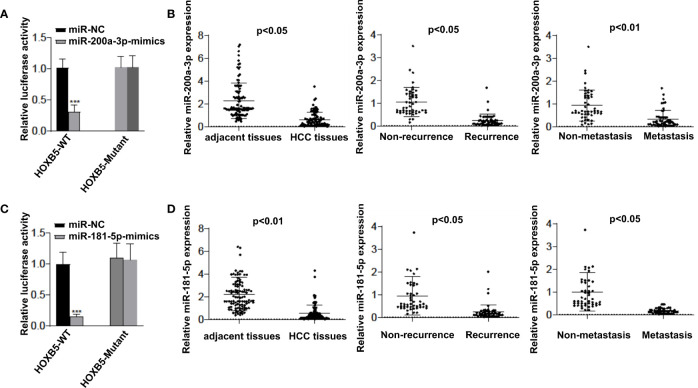
miR-200a-3p and miR-181-5p upregulates HOXB5 expression to contributes HCC progression. **(A)** Luciferase reporter assay confirmed that miR-200a-3p mimic could successfully made a dramatic reduction in the relative luciferase activity when HOXB5 3′ UTR sequences containing WT miR-200a-3p binding sites compared with HOXB5 3′ UTR sequences containing mutant miR-200a-3p binding sites; **(B)** Expression of 200a-3p in 100 pairs of HCC tissues and adjacent non-tumor tissues was evaluated by qRT-PCR. Data are mean ± SD, n=100;** (C)** Luciferase reporter assay confirmed that miR-181-5p mimic could successfully made a dramatic reduction in the relative luciferase activity when HOXB5 3′ UTR sequences containing WT miR-181-5p binding sites compared with HOXB5 3′ UTR sequences containing mutant miR-181-5p binding sites; **(D)** Expression of miR-181-5p in 100 pairs of HCC tissues and adjacent non-tumor tissues was evaluated by qRT-PCR. Data are mean ± SD, n=100. ***p < 0.001.

However, the regulatory mechanism of HOXB5 upregulation in human HCC remains unclear. The aberrant expression of miRNAs has been considered to be a critical player in HCC progression (15, 16). Considering that HOXB5 is a master target of multiple cancer-related miRNAs (12, 13), we raised the question of whether certain miRNAs regulate HOXB5 expression in HCC progression. To test our hypothesis, we screened the predicted potential miRNAs that could bind with HOXB5 promoter regions. Then, we chose to further study miR-200a-3p and miR-181-5p because these two miRNAs showed the most power in regulating HOXB5 expression when HepG2 cells were treated with inhibitors of certain miRNAs. The luciferase reporter assay confirmed that miR-200a-3p and miR-181-5p mimic could successfully make a dramatic reduction in the relative luciferase activity with HOXB5 3′-UTR sequences containing WT miR-200a-3p or miR-181-5p binding sites (**Figures 5A, C**). To further evaluate the role of miR-200a-3p and miR-181-5p in HCC progression, we tested the expression levels of miR-200a-3p and miR-181-5p in 100 pairs of HCC tissues and adjacent non-tumor tissues. The expression of miR-200a-3p and miR-181-5p was dramatically quenched in HCC tissues than in their adjacent non-tumor tissues (**Figures 5B, D**). Furthermore, miR-200a-3p and miR-181-5p levels were significantly lower in recurrence patients and in metastasis patients than in patients without recurrence or metastasis (**Figures 5B, D**). Collectively, these data suggested that decreased miR-200a-3p and miR-181-5p expression in HCC is a key regulator to upregulate HOXB5 levels to contribute to HCC progression.

## Discussion

In the present study, we found that HOXB5 is markedly upregulated in HCC tissues compared to paired-adjacent noncancerous controls, which was consistent with other research groups (17, 18). In addition, we demonstrated that overexpression of HOXB5 was associated with tumor size, tumor-nodule metastasis, TNM stage, and relatively unfavorable OS and DFS. By using overexpression and downregulation of HCC cell lines of HOXB5, we demonstrated that ectopic expression of HOXB5 in HepG2 cells promoted the capacity of cell growth and clonogenicity, while the inhibition of HOXB5 in Hep3B cells decreased the proliferation and clonogenicity potential *in vitro* by CCK8 and colony-forming assays. In addition, HOXB5 also promoted cell migration *in vitro* by transwell experiment. Mechanism studies elucidated that upregulation of HOXB5 is regulated by miR-200a-3p and miR-181-5p. Transfection of miR-200a-3p or miR-181-5p mimics in HepG2 cells blocked the cell proliferation and metastasis regulated by HOXB5, while overexpression of the 3′-UTR mutant HOXB5 abolished the suppressive effect of miR-200a-3p or miR-181-5p. In addition, we found that HOXB5 triggers HCC progression *via* direct transcriptional activation of EGFR. Both the clinical data and the experimental results suggested that HOXB5 plays an important role in HCC progression.

In recent years, anti-EGFR therapy has achieved tremendous success in solid tumors of the digestive system, such as colorectal cancer (4). Previous studies provided solid evidence that anti-EGFR monoclonal antibodies and EGFR tyrosine kinase inhibitors are promising treatment methods for HCC (19–21). However, anti-EGFR therapy failed to meet expectations in HCC treatment. So far, the underlying mechanisms remain elusive. Thus, targeting the upstream regulators of EGFR brings the attention to HCC therapy. Our study found that EGFR is a key transcriptional target of HOXB5. Knockdown of EGFR extensively suppressed HOXB5-mediated HCC progression, whereas overexpression of EGFR rescued the progression of HCC mediated by HOXB5 inhibition. Importantly, we also confirmed the strong correlation between EGFR and HOBX5 expression in human HCC tissues, and patients with positive co-expression of HOXB5 and EGFR showed the poorest OS. The ChIP assay further demonstrated that HOXB5 binding was indeed enriched in promoter regions of EGFR between −790 and −625 in both HCC cell lines and human HCC tissues. All these findings suggested that HOXB5-facilitated HCC progression partly depended on regulating EGFR expression. However, no clues were available on why HOXB5 is upregulated during HCC progression. Given that dysregulation of miRNAs is a key characteristic of HCC, and HOXB5 is also a master target of multiple cancer-related miRNAs (12, 13), we determined whether upregulation of HOXB5 in HCC is a result of dysregulation of certain miRNAs. We found that miR-200a-3p and miR-181-5p were the most powerful miRNAs in regulating HOXB5 expression in HCC cells. Downregulation of miR-200a-3p frequently occurred during the HCC progress and acted as a prognostic factor for HCC patients (22, 23). The luciferase reporter assay confirmed that miR-200a-3p and miR-181-5p mimics could successfully reduce the relative luciferase activity with HOXB5 3′-UTR sequences containing WT miR-200a-3p and miR-181-5p binding sites. Moreover, the expression of miR-200a-3p and miR-181-5p was dramatically quenched in HCC tissues compared to their adjacent non-tumor tissues. In addition, miR-200a-3p and miR-181-5p levels were significantly lower in recurrence patients and in metastasis patients than in patients without recurrence or metastasis. These data suggested that decreased miR-200a-3p and miR-181-5p expression in HCC are key regulators to upregulate the HOXB5 level.

Collectively, our study reported a role for HOXB5 in HCC progression. HOXB5 promoted HCC proliferation and progression by direct transcriptional activation of EGFR. In addition, we found that miR-200a-3p and miR-181-5p upregulated HOXB5 expression to promote HCC proliferation and progression. Thus, HOXB5 is a prognostic biomarker in human HCC, and targeting miR-200a-3p or the miR-181-5p/HOXB5/EGFR signaling pathway may provide evidence for the treatment strategies for HCC.

## Data Availability Statement

The original contributions presented in the study are included in the article/supplementary material. Further inquiries can be directed to the corresponding author.

## Ethics Statement

The studies involving human participants were reviewed and approved by the Ethics Committee of the First Affiliated Hospital of Xi’an Jiaotong University, and written informed consents were signed by participants prior to enrolling in this study. The patients/participants provided their written informed consent to participate in this study.

## Author Contributions

HX and WL drafted the manuscript, participated in research design, conducted experiments, and validated the data. YL, PL, FM, ML, and SK participated in research design, conducted experiments, contributed to the writing of the manuscript, discussed data, and supervised the study. All authors performed data analysis and interpretation and read and approved the final manuscript.

## Funding

The project was supported by the Key Research & Development Program of Shannxi Province (No. 2019SF-210).

## Conflict of Interest

The authors declare that the research was conducted in the absence of any commercial or financial relationships that could be construed as a potential conflict of interest.

## Publisher’s Note

All claims expressed in this article are solely those of the authors and do not necessarily represent those of their affiliated organizations, or those of the publisher, the editors and the reviewers. Any product that may be evaluated in this article, or claim that may be made by its manufacturer, is not guaranteed or endorsed by the publisher.

## Abbreviations

HCC, hepatocellular carcinoma; EGFR, epidermal growth factor receptor; HOX, homeobox; MMP, matrix metalloproteinase; EMT, epithelial-to-mesenchymal transition; miRNAs, microRNAs; OS, overall survival; DFS, disease-free survival; FBS, fetal bovine serum; PVDF, polyvinylidene fluoride; RT, room temperature.
